# Computational Modeling of the Value Co-Creation Process in Customer Service: An Application of the NK Model

**DOI:** 10.3389/fpsyg.2022.868803

**Published:** 2022-05-17

**Authors:** Xi Li, Tomoki Sekiguchi, Jiunyan Wu, Qiongwei Ye

**Affiliations:** ^1^Nanchang Institute of Science and Technology, Nanchang, China; ^2^Graduate School of Management, Kyoto University, Kyoto, Japan; ^3^Head of People & Organization Talent and Leadership of Siemens, Beijing, China; ^4^Yunnan University of Finance and Economics, Kunming, China

**Keywords:** value co-creation process, computational simulation, NK model, customer engagement behavior, dynamic interaction

## Abstract

There has been an increasing interest to explore and gain knowledge about customer engagement behavior among academia and practitioners. Particularly, the value co-creation process in customer services is essential to explore the interaction structure. In this study, we applied the computational simulation of the NK model to identify the value co-creation process between service employees and customers in the service context. To specifically explore the dynamic interaction among them, we identified what kind of service is provided for what type of customers and when service performance improves according to the degree of interaction between service employees and customers. The simulations show that the greatest service value can be achieved when employees and customers jointly perform local search (90%) and long jump (10%). However, if both employees and customers jointly perform local search only, the service value can be stuck in a local optimum. In cases where employees and customers make their independent improvement, either through local search or long jump, the overall service value varies depending on the complexity of interactions between employees and customers. For example, the improvement in service value is the worst when employees and customers make long jumps at independent timings in high complex interactions. Our computational simulations offer visible experimental-based insights into understanding the value co-creation process with customers and promising results for customer service studies.

## Introduction

Over the past decades, as the global economy has become more dependent on services, customer service has become increasingly important for attracting and retaining consumers in both the manufacturing and service industries. Delivering high-quality customer services has been considered a vital strategy for business success and subsistence in a competitive marketing environment (Parasuraman et al., 1985; Dukes and Zhu, 2019; Ingene et al., 2020; Wang et al., 2020a,b). In the Internet era, with the development of omnichannel marketing, a bunch of online retailers adopted additional offline channels to provide better shopping environments. Despite the efforts from both academia and the industry in seeking ways to enhance customer services, the outcomes of customer services differ depending on the service contexts, such as online (mobile channel or PC channel) and offline channels (Sun et al., 2017; Güntürkün et al., 2020; Li et al., 2021a). For example, in tangible product-based omnichannel (e.g., automobile dealers and sports retailers), customer services have been recognized as those additional services that are provided to customers above and beyond the basic benefits of products (Bowen et al., 1989; Li et al., 2021b). Meanwhile, in service-based contexts (e.g., sports clubs), service value is created during the process of customer service encounters (Teng, 2019). Generally, service-based contexts, especially the interaction service contexts, differ from business activities that involve tangible products, with one of the distinct features of service being the process of creating value via service employee interaction with customers (Groth et al., 2019). Previous research identified that employee satisfaction significantly impacts customer satisfaction (Li et al., 2018). Specifically, frontline service employees have been frequently the foremost contact customers have with retailers and service providers (Zeithaml et al., 1996; Brodie et al., 2013; Prentice et al., 2019; Behnam et al., 2021). Conceptualized under the service-dominant logic, customer value is created by customer engagement with the service provider (Vargo and Lusch, 2017). In this regard, growing attention has been paid to customer engagement in the recent decade (Van Doorn et al., 2010; Ho and Chung, 2020; Liu et al., 2021). However, research on the dynamic interaction between service employees and customers is still limited (Argo and Dahl, 2020; Li and Katsumata, 2020; Zhang et al., 2020).

Essentially, research on customer engagement behavior, such as value co-creation activities, has become one of the top research priorities in the past decade in the management and marketing sector (Verhoef et al., 2010; Grisemann, 2012; Behnam et al., 2021). More broadly speaking, a better understanding of the process of value creation in customer service via the interaction between employees and customers will effectively benefit the recruitment of employees engaged in customer service, employee capacity building, as well as other aspects of talent management. However, there is little evidence regarding how to best gauge the value of the co-creation process between service employees and customers in the retailing and hospitality settings.

Ensuring outstanding customer service quality (SQ) has been widely recognized as a vital business requirement (Menguc et al., 2020). Specifically, based on the service-profit chain, the service employee performance is considered to directly lead to creating financial benefits and enhancing corporate competitiveness (Heskett et al., 1994). A few studies in the literature have been based on the service-profit chain (Heskett et al., 1994) to examine the relationship among customer services, customer loyalty, and financial gains (Liu and Qu, 2019; Strydom et al., 2020). However, the number of studies that explore the dynamic nature of the value co-creation process between service employees and customer engagement is quite limited. Therefore, to identify the process of the value co-creation and service interactions between employees and customers' engagement, this study applied Kauffman's NK model and rugged fitness landscape (Kauffman, 1995; Baumann et al., 2019), which are originally derived from the field of evolutionary biology. More specifically, we applied computational simulation of the NK model to explore what kind of service is provided for what type of customers.

## Literature Review

### The Concept of Customer Service

Customer service, also referred to as customer service performance or other variants of this terminology, can be defined as any activity of employees directed toward affecting service quality (Rogelberg et al., 1999; Ryan and Ployhart, 2003; Groth et al., 2019). The performance of service employees often varies from producer to producer, time to time, and individual to individual (Shostak, 1977). Customer service pertains to a multi-dimensional construct involving a large variety of emotional, behavioral, and affective components, ranging from financial performance to customer experience and relationship building (Wang and Yi, 2020). The core characteristics include intangibility, heterogeneity, and simultaneity. More specifically, service intangibility means that services cannot be measured, counted, or tested as tangible objects, including services that are heterogeneous. Service simultaneity means that production and consumption are inseparable, and they also involve customer participation.

More recently, more studies are focusing on customer engagement in providing services. Chae (2012) recognizes that service is interactive or related, the value of service is co-created or co-produced by service employees and customers, and that production and consumption are simultaneous with an ambiguous boundary. Groth et al. (2019) focus on the influence of customer service on customer engagement in providing service. Geng et al. (2022) from the perspective of consumers further identifies the role of consumer trust in corporate innovativeness.

Existing evidence shows that the service-dominant logic should consider the above-mentioned nature of service when applying it to the commercial activities accompanying merchandise and products (Hartwig et al., 2021; Tran et al., 2021). Following this logic, services are no longer considered as output, but as the process, pattern, and benefit for value creation through an exchange (Groth et al., 2019; Lalicic and Weismayer, 2021). Since service is inherently interdependent and involves multiple entities, the process of service is complex and with high uncertainty, and therefore the value of service is hard to predict.

From the customer service perspective, the quality of the employee–customer interaction plays a critical role in service performance and is recognized as a “moment of truth.” However, the process is not easy to elucidate (Groth et al., 2019). Therefore, the main purpose of this study is to present a dynamic model to explore the service value co-creation process through the employee–customer interaction by adopting the NK model.

### Employee and Customer Value Co-Creation

Value co-creation refers to the process during which consumers take an active role and co-create value together with the company through direct and indirect collaboration (Kohler et al., 2011). Generally, value co-creation includes the roles of engagement, interaction, and experience (Bendapudi and Leone, 2003). Value co-creation was fueled by the studies of Vargo and Lusch (2004) on co-creative service-dominant logic in the context of marketing. In past decades, theoretical and empirical attempts have blossomed in different research fields, such as the earlier co-production (Prahalad and Ramaswamy, 2000), supervisor and student value co-creation (Li et al., 2018), customer value co-creation behavior in the purchase phase (Bu et al., 2022), leader and member co-production (Duan et al., 2022), and value co-creation between service providers and customers (Sheng et al., 2022).

Previous research on value co-creation between service employees and customers has been well examined in multi-context, such as retailing, hospitality, and social media (Wang et al., 2020a,b; Zhang et al., 2020; Yi et al., 2021a; Bu et al., 2022). However, the process of service providing is often influenced by the cultural background of consumers (Sun et al., 2017; Yi et al., 2021b), corporate reputation (Geng et al., 2022), and peer influence (Sun et al., 2021). Accordingly, the complicated dynamic nature of value co-creation between service employees and customers has not been well explored. In this study, we aim to apply NK Model to simulate the dynamic process of value co-creation between service employees and customers.

### NK Model of Rugged Fitness Landscapes

Kauffman's NK model (Kauffman, 1995) is a framework for estimating the influence of interdependencies within an evolutive system. In this model, the subject of a study is usually taken as a total of N components. For example, the process of an enterprise making decisions in daily operation can be regarded as a set of N decisions, and those results directly determine the performance of the enterprise. This is the same as in the field of evolutionary biology (where the NK model was originally conceived), which considers the characteristic gene of a given organism to be a set of N choices related to morphology and traits and expresses them as actual morphology and traits. Besides, the performance caused by N decisions is credited, on average, to be affected by K's other decisions (Kauffman and Weinberger, 1989). Such a complex combinatorial optimal process can be characterized as “uphill walks on rugged fitness landscapes,” where F is uniformly distributed on values 0 or 1, and they can be bidirectionally or unidirectionally interdependent. Contrary to the assumption that each component plays a strong and independent role in the consequence, as Kauffman and Weinberger (1989) observed, in the case of K = 0, trivial components do not interact with each other, and there exists only one maximum. Generally, the interaction between components is weak when the K value is small, and the interaction is strong when the K value is large. The complex process, therefore, makes the result hard to predict.

From the perspective of environmental adaptability, the output of the NK model is called rugged landscape or rugged fitness landscape (Baumann et al., 2019). The NK model shows an important attribute that as the parameter K increases, the “ruggedness” of the NK landscape ranges from a single-peaked “Fujiyama” landscape to a multi-peaked “badlands” landscape. For instance, if the value of K is 0, the landscape is as simple as “Fujiyama.” However, when the value of K becomes large, the degree of ruggedness increases, and the landscapes turn into complex mountainous terrains. Moreover, as K increases, distances to local optima on such “badlands” landscape become shorter. In the field of evolutionary biology, when a gene of N components mutates and takes on different forms, adaptation to the environment will work in the gene's favor, while failure to adapt will work against it. In a rugged landscape, the degree of conformity to such an environment is compared to the height of the mountain. According to the results of N decisions, in cases where the theory is applied to the performance of a manufacturer and service provider, if it is strongly compatible with the business environment and meets customer needs, the landscape can be interpreted as a high mountain (performance improvement) or otherwise a low mountain (performance decline).

In the literature of management, following the study of Levinthal (1997) who applied the NK model to organizational adaptation, this model has been used in various management topics, such as organizational decision making (Billinger et al., 2014) and open innovation (Almirall and Casadesus-Masanell, 2010). In the metaphor of rugged landscape that relates performance to a mountain in business management, climbing a mountain equals improving performance, while going down a mountain denotes a drop-off in performance. Both enterprises carrying out business and employees providing services use management and operations to improve performance, such as customer satisfaction and financial performance. Their performance can be interpreted as companies and employees exploring a mountain with poor visibility in a quest to reach the peak.

An important assumption to note is that companies and employees in the rugged landscape cannot grasp the entire picture according to the principle of bounded rationality. Under this assumption, if the K value is small and each component contributes to performance independently, it is possible to maximize performance by climbing a large mountain without seeing the full picture of the rugged landscape. In other words, for a company or employee, it is relatively easy to understand what should be done to improve performance, as long as one climbs up the mountain step by step while repeatedly adjusting various components. However, when the large value of K and the rugged and complex terrain make it impossible to grasp the whole picture, that is, when the vision is not good, one must grope for the right decisions and actions to reach the highest peak. Then it would be difficult for a company or employee to clarify what to do to improve performance. This is consistent, as described earlier, with the complexity of the service process (one of its essential characteristics is co-creating value through employee–customer interaction) and the difficulty in understanding how to maximize service performance.

In the NK model and rugged landscape, local search and long jump are frequently adopted to classify decisions and actions made by the active subjects (Billinger et al., 2014). Local search refers to the small movement by changing a few components, which can be compared to a step-by-step climbing to the mountain top while repeatedly improving the *status quo* (Billinger et al., 2014). The other concept, long jump, refers to moving significantly by changing most of the N components, likened to the activity of exploring distant mountains in search of great change and innovation (Levinthal, 1997). The advantage of local search is that one can move toward the top of the mountain that one currently climbs, but the disadvantage is that higher performance cannot be expected because it may be a small mountain. That is, after reaching a local peak, one can no longer expect performance improvement because the only option is in the downward direction. On the other hand, while the long jump boasts the advantage of increasing the likelihood of finding large mountains and maximizing performance, that is, reaching global peaks, it also entails the risk of not being able to find a high mountain due to poor visibility. Therefore, one of the research patterns using the NK model and rugged landscape is how to combine local search and long jump under a given rugged landscape to maximize performance and fitness to the environment.

## The Application of the NK Model to Customer Service

In this research, the NK model was applied to the process of co-creating service value through employee–customer interaction. In general, each of the N elements has (0, 1) option, and performance is the sum of the results derived from the choice of N elements. Therefore, the value of the services in this study is viewed as a combination of tangible (commodities, etc.) and intangible elements (behavior, skills, etc.) in the employee–customer interaction. Importantly, some of the N elements that determine the service value, such as how employees deliver goods and deal with customers, as well as factors concerning customer response and cooperation actions, are coded in the binary system. In addition to service providers and customers, Chae (2012) added the location of service to the model as a crucial component, but for simplicity, the element of location is omitted in this research.

The number of N depends on the characteristics of customer service, but the value of K and the pattern of the specific interaction are determined according to the degree of interaction between elements and the interaction between behaviors and skills within employees and customers, as well as the characteristics of interaction between employees and customers. In general, since the interaction pattern can be both unidirectional and bidirectional as in the example of the two independent elements A and B, a matrix is used to represent the four patterns of A → B, B → A, and A⇔B. Specifically, this research assumes that *N* = 10, the first five factors are on the employee's side, and the last five factors are on the customers' side.

**Figure 1:**
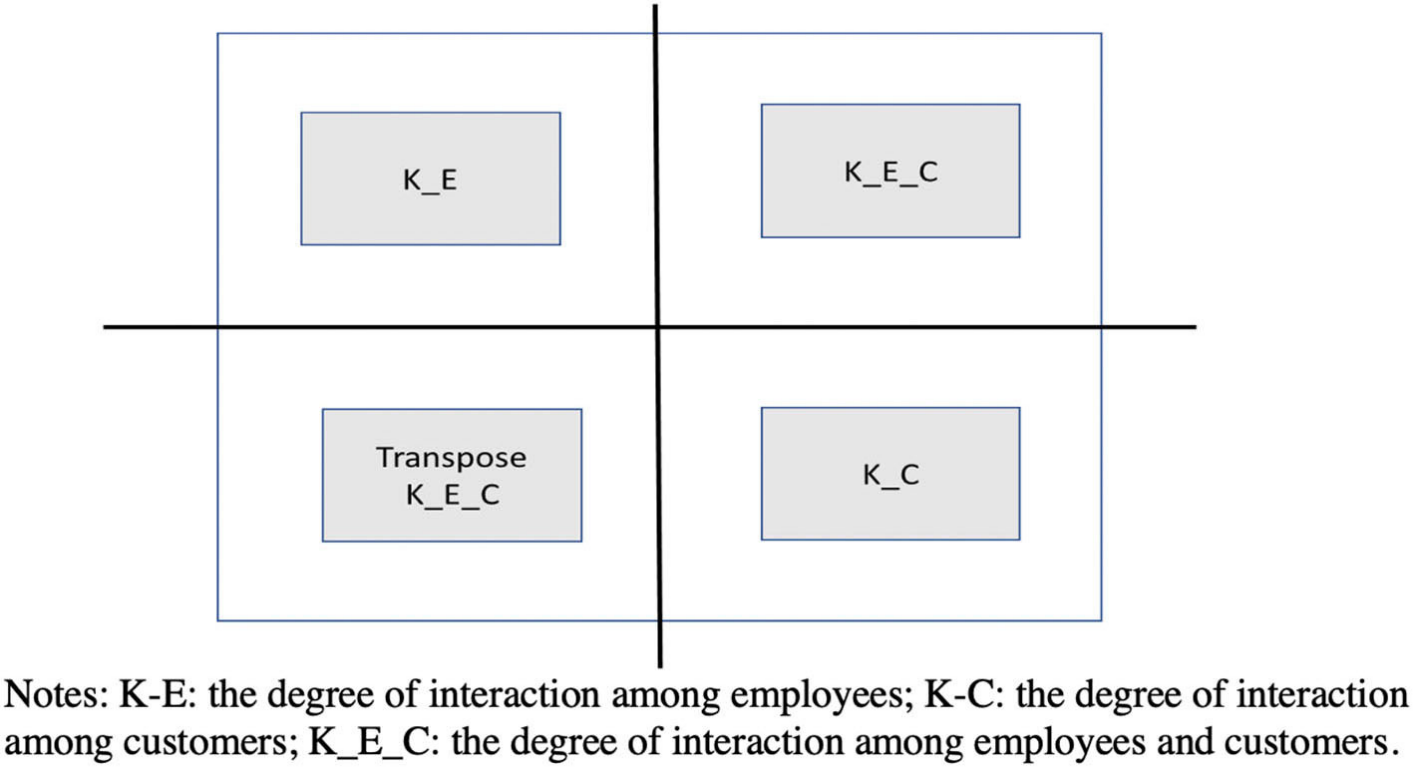
Employee-customer internal and external interaction matrix. K_E, employees; K_C, customers; K_E_C, interactions.

**Figure 2:**
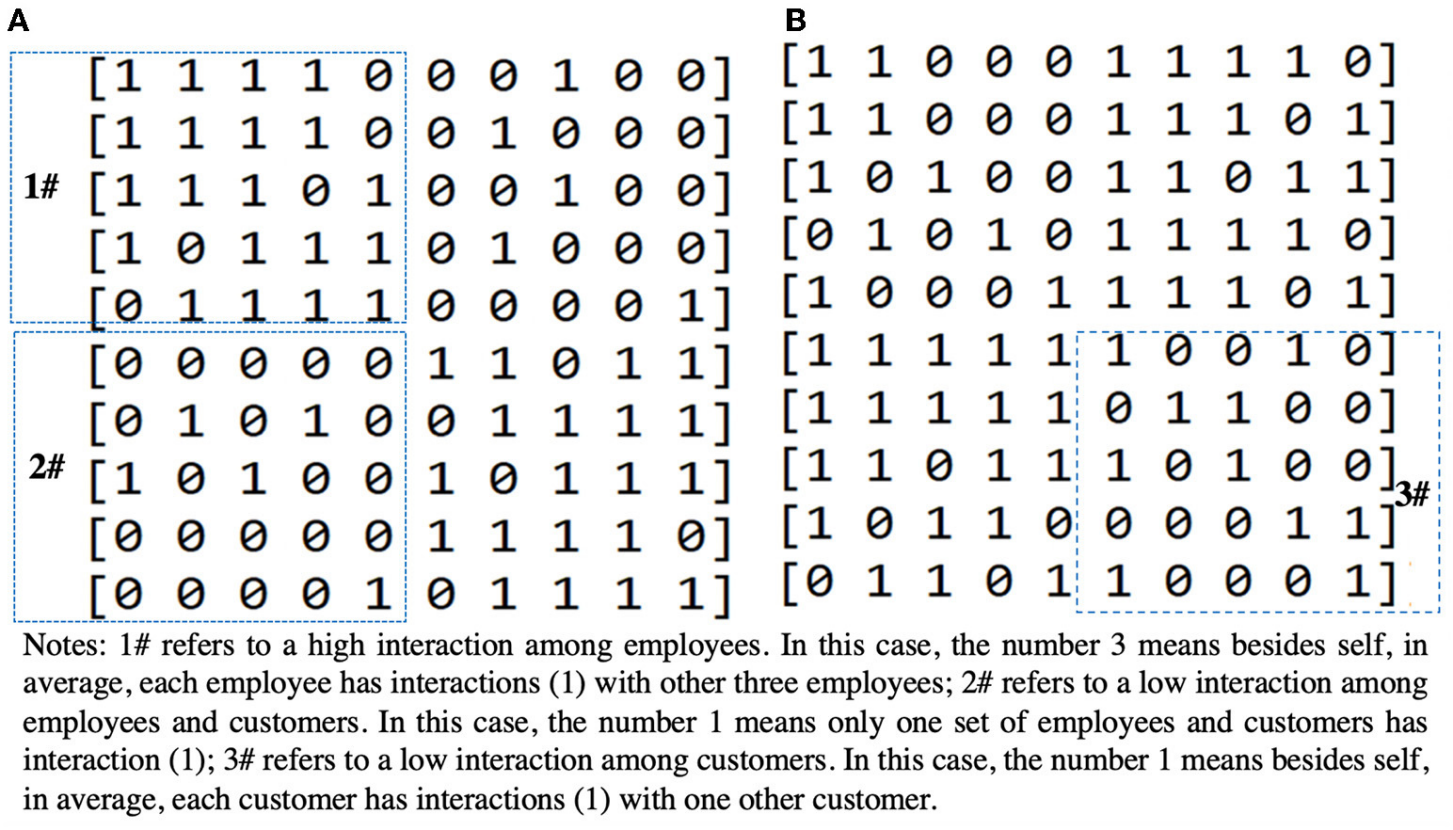
**(A)** Matrix of NK model, high/high/low = 3/3/1, **(B)** Matrix of NK model, low/low/high = 1/1/4.

As shown in Figure 1, the quadrant of the matrix represents an area revealing the pattern of inter-element interactions within employees, an area that indicates the pattern of inter-element interactions within customers, and an area that shows a pattern of inter-element interactions that span employees and customers. Set “1” to one particular component if it affects another component, and “0” if it does not, and the direction of interaction is symmetric/asymmetric in the diagonal region. For example, in the matrix where *N* = 10, K = 4, and an interaction degree is high/high/low = 3/3/1, as shown in Figure 2A, any element that affects any other element is determined at random. On the other hand, in the matrix where *N* = 10, K =5, and low/low/high = 1/4/4, any element that affects any other element is determined at random, as shown in Figure 2B.

The rugged landscape of service value creation is determined by the characteristics of this matrix, that is, the employee's service provision, the customer's response to it, and the interaction of factors involved. When employees and customers change the element respectively (0→ 1 or 1→ 0), the increase or decrease of the value of service depends on the ruggedness of the landscape.

According to the aforementioned metaphor of exploration in the mountain with poor visibility, employees and customers co-create service value through the “moment of truth,” but little is known about what combination of elements maximizes the service value, namely, the location of the highest peak on the rugged landscape. Starting from this *status quo*, and changing the way the elements are combined, both sides aim to improve the service value by jointly exploring the mountainous area. The higher the interdependence between employees and customers, the more difficult it is to enhance service value only by their own choices.

In such an environment, both employees and customers view local search and long jump as two types of decision making and action. One method in the case of local search involves changing a few controllable elements to observe the outcome. The following information shows some behavior changes on the employee's side, such as a slight improvement in the content or method of service. It is the same on the customer side. The following transition shows service improvement through local search from the employee's side.

10100 → 10000

Long jump refers to the act of simultaneously changing a large number of elements out of multiple controllable ones. In the following case, we aim, on the employee's side, to dramatically improve the value of service through creativity and innovation by substantially changing the content and method of service provided. It is the same on the customer side. The following transition shows attempts at radical service innovation through a long jump from the employee's side.

10100 → 01111

The purpose of this exploratory research using the NK model is to realize the improvement of service value by combining local search and long jump between employees and customers in the aspect of customer service.

## Computer Simulation

The communication simulation was performed on the above-mentioned NK model set, which consists of service values co-created by employees and customers. In practice, we used a modified form of Python code for the NK model provided by Workiewicz (2019). First, the rugged landscape was created using the K value and the pattern of interaction under the condition of *N* = 10. Then, based on the resulting rugged landscape, a simulation where employees providing service and customers responding to service were combining local search and long jump was conducted. By altering the K value and the pattern of interaction and changing the combination of local search and long jump, respectively, on the employee's side and customer's side, 500 tests were repeatedly conducted on the computer to explore how to improve the performance.

In the studies related to customer service, various situations and conditions concerning service characteristics, customer needs, and employee–customer interaction have been considered. Since this study is exploratory, on the setting of *N* = 10 (employee 5 + customer 5), the characteristics of customer service and the status of employee–customer interaction were set as follows: interdependence of the elements that make up the service: 0–3, interdependence of customer needs: 0–3, and employee–customer interdependence: 0–4.

Next, for the setting of local search and long jump, two cases were assumed: employees and customers separately performing local search and long jump at a constant frequency, and employees and customers jointly (simultaneously) performing local search and long jump at a constant frequency. Simulations were carried out with changing frequencies of occurrence.

In the simulation, service employees and customers, starting from any point of the given rugged landscape, independently or collectively performed a local search or long jump, based on the various conditions set, until the value of the service increased. Two cases were set: one for local search only and the other with 90% of local search and 10% of long jump (but at random timing).

Improvement in service value signified the completion of one step, where the simulation proceeded to the next search. After 500 steps, a record of how the service value had increased was kept. In addition, we repeated 1,000 trials and obtained the result of how the value increased on average. A summary of the simulation results is shown in Table 1, which records the service value after 500 steps (the average value after 1,000 trials/attempts at simulation) for each of the four patterns of service characteristics, customer needs, and employee–customer interaction.

**Table 1:** Results of computer simulation (service value after 500 steps).

**Pattern**	**Local search**	**Long jump**	**(Emp/cus) local/local**	**(Emp/cus) jump/local**	**(Emp/cus) local/jump**	**(Emp/cus) jump/jump**
L/L/L (1/1/1)	0.913	0.960	0.855	0.842	0.846	0.831
L/L/H (1/1/4)	0.851	0.917	0.739	0.708	0.706	0.689
H/H/L (3/3/1)	0.868	0.931	0.910	0.900	0.901	0.894
H/H/H (3/3/4)	0.812	0.896	0.784	0.750	0.741	0.718

**Figure 3:**
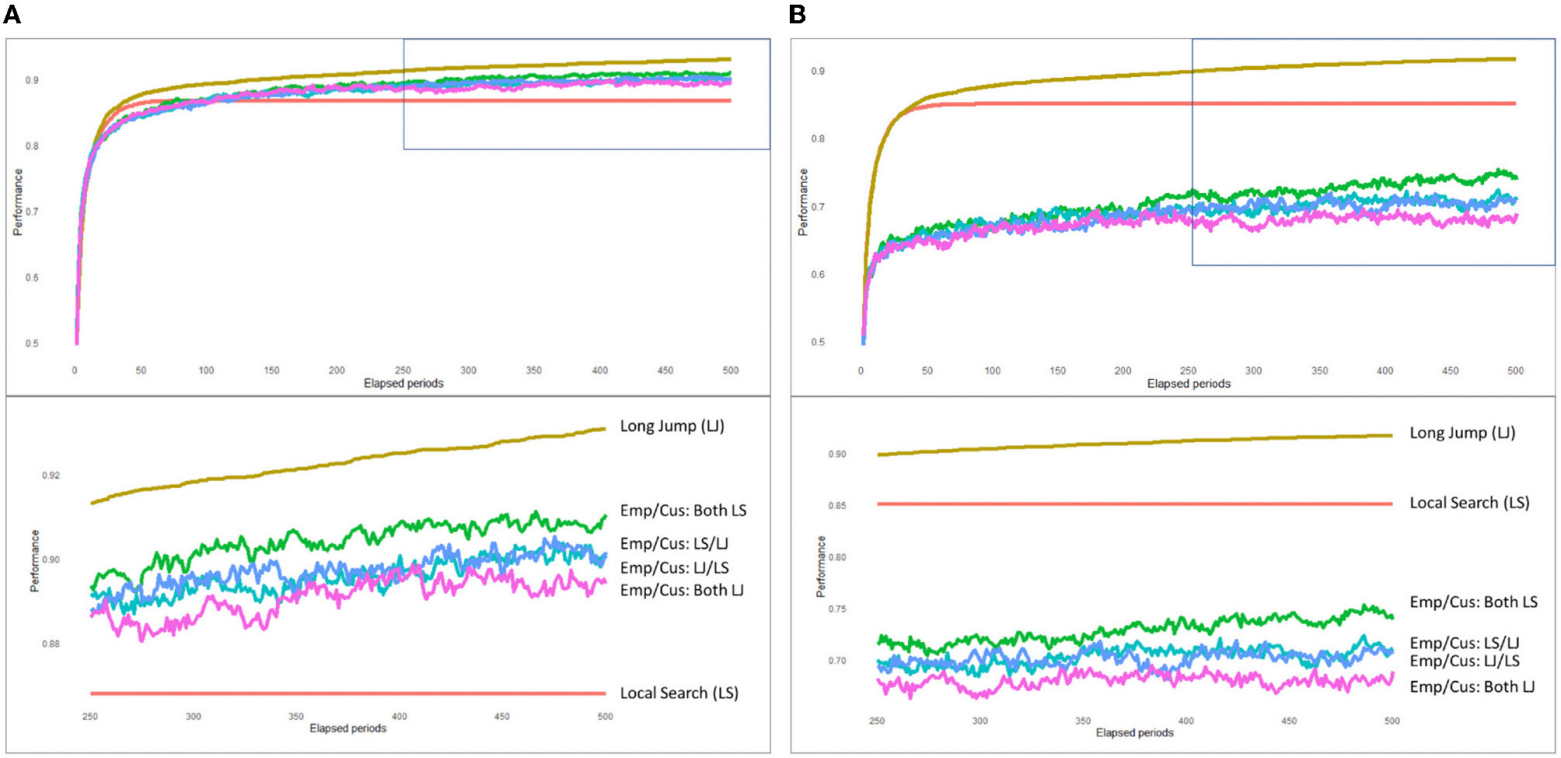
**(A)** L/L/H (low/low/high) case. **(B)** H/H/L (high/high/low) case.

For L/L/H(low/low/high) and H/H/L(low/low/high) of the four patterns, the average trend of service value improvement at 500 steps is shown in Figures 3A,B. This graph shows that service value, though low at the start, was gradually improved by local search or long jump of employees and customers, and in some cases, its improvement reached the plateau at a certain point.

We used computational modeling to simulate the value co-creation process in customer service and observed the following results. First, the greatest service value was achieved when employees and customers working together on local search and long jump accounted for 10%. By performing a local search that targets improving service value and by performing long jumps together at the right timing, there is a high probability of achieving a breakthrough in service value (finding another high mountain). However, in the case where employees and customers only jointly performed the local search, the increase in service value was not observed early in the steps and the plateau was reached. This was probably because when the local peak was reached early in the steps, it was impossible to get out of it without a long jump. In other words, relying solely on the joint local search of employees and customers can only increase the value by improving extant services.

Second, service content and customer needs are not over-complex, but service value cannot be fully improved when employees and customers independently conduct the local search or long jump or if interdependence between the service and the customer needs greatly influences service value. Particularly, in cases where employees and customers made long jumps at independent timings, improvement in service value was the worst. This is because, in many cases, even if employees and customers can achieve value improvement in controllable parts, the addition of those parts does not necessarily lead to an overall improvement in the service value.

Third, the case where employees and customers concurrently and jointly conducted the local search or long jump is considered to have a significant effect on increasing service value. Specifically, actions including long jump at a rate of 10% resulted in the greatest improvement in service value. In contrast, the interdependence between the service of employee and customer needs had a low impact on the service value. In face of the high and complex interdependence of service content and customer demand, the improvement in service value was the worst when employees and customers only jointly conducted local search. This was probably because, as mentioned earlier, the local peak was reached early in the steps, and it was not possible to get away from it without a long jump.

Finally, service value is best achieved when employees and customers combine local search with a long jump. When employees and customers performed an independent local search or long jump, they got the same results as in other cases. However, with repeated steps, it resulted in a continued gradual increase in service value. This may be because the degree of ruggedness was high from the perspective of service content and customer demand, and when employees and customers acted independently, many elements help them find higher points in the mountain.

## Discussion and Academic Implications

This study focuses on the co-creation of service value between employees and customers, which is complex and difficult to understand intuitively or theoretically. Therefore, Kauffman's NK model and computer simulation using rugged landscape were applied for the explorative study.

Even after the results were logically interpreted, we still obtained inconsistent results and observed unexpected results, such as the difficulty to detect any discovery without computer simulation. For example, as in Figures 3A,B, the effect of local search jointly performed by employees and customers varied greatly depending on the situation, which was unexpected and difficult to assume before the simulation. Thus, computer simulation (combining the NK model and rugged landscape) was effective in the study of complex and uncertain service value creation. Using simulation models, this study revealed the dynamic process of co-creating value between employees and customers, which made up for the lack of cross-sectional data research or questionnaire methods, goes beyond the traditional static theory (Vancouver et al., 2020), and provides a dynamic theory of co-creating value between employees and customers.

Since computer simulation was conducted in an exploratory manner with only limited types of cases under a small number of setting conditions, it remains challenging to obtain deep insights into the co-creation of service value by this mere result. However, some theoretical and practical implications can be derived. First, this study confirmed that it is vital for employees and customers to keep pace in the creation of service value. More specifically, when it comes to improving services and changing customers' needs, the service value cannot be improved even if employees and customers work differently. Instead, it is effective to improve service value when employees and customers synchronously make local search, that is, fine-tune (improvement) on extant services and needs, and when they make long jump, adventures, and make decisive changes in service or needs accordingly. To realize such activities that align employees and customers, facilitating communication between employees and customers and fostering their mutual understanding and common awareness are recommended. Therefore, this study reveals the boundary conditions for realizing the maximum co-creation value between employees and customers and provides new knowledge on the combination of factors for co-creation value between employees and customers.

### Practical Implications

First, corporate managers should provide customers with opportunities for value co-creation, which can promote customer experience and interactive co-creation activities. Enterprises should consider the relevant factors for co-creating value with customers in their organizational design, service process design, etc., to provide customers with opportunities for value co-creation and to realize the co-creation of value between employees and customers. For example, Xiaomi, a well-known mobile phone company in China, pioneered the use of the Internet model to attract customers to participate in the development and improvement of Xiaomi mobile phones. This kind of co-creation activity between employees and customers not only develops products that customers demand, but also cultivates a large number of fans of Xiaomi's new products.

Second, corporate managers should reward employees whose service provision processes co-create value with customers, encourage employees to actively cooperate with customers, and mobilize employees' enthusiasm for co-creating value with customers, to realize the co-creation of value between employees and customers.

Third, corporate managers should facilitate activities that co-create value to increase the authorization of customers and promote interaction between employees and customers. Under the policy of organizational support for co-creation of value, corporate managers should increase the authorization of customers and increase customers' sense of control over the value co-creation process, so as to realize the co-creation of value between employees and customers.

### Limitations and Future Directions

There are two limitations in this study. First, the appropriate proportion of long jump has not been discussed in this simulation, and it should be addressed in the future research. When local search is implemented, that is, only the extant services of employees are improved and only the extant needs of customers are slightly adjusted, the local peak will be reached in the early stage, and the improvement of service value will enter a plateau state. To achieve a breakthrough in improving service value, it would be useful to make a long jump at a certain point at a certain frequency, that is, a substantial change in service and a corresponding alteration in customer demand. As mentioned earlier, if employees and customers are not acting in an orchestrated manner, the results will be unfavorable, and the value will probably deteriorate. Second, the present study focused on a small number of simple cases for the co-creation of service value. In future research, by refining the model of co-creation of service value, appropriately applying it to the NK model, and examining a large number of more complex cases, it is expected to make promising but rational discoveries that are difficult to be explored without utilizing the NK model and rugged landscape.

## Data Availability Statement

The raw data supporting the conclusions of this article will be made available by the authors, without undue reservation.

## Author Contributions

TS contributed to the writing of the first draft. XL contributed to enriching the first draft. JW contributed to the empirical work, analysis of the results, and writing of the first draft. QY contributed to the overall quality and supervised the literature organization and empirical work. All authors discussed the results and commented on the manuscript. All authors contributed to the article and approved the submitted version.

## Funding

This research was supported by JSPS KAKENHI (grant number 19K21692), Kunming E-commerce and Internet Finance R&D Center (KEIRDC [2020]), and the Prominent Educator Program (Yunnan PEP [2018]11).

## Conflict of Interest

JW was employed by Siemens. The remaining authors declare that the research was conducted in the absence of any commercial or financial relationships that could be construed as a potential conflict of interest.

## Publisher's Note

All claims expressed in this article are solely those of the authors and do not necessarily represent those of their affiliated organizations, or those of the publisher, the editors and the reviewers. Any product that may be evaluated in this article, or claim that may be made by its manufacturer, is not guaranteed or endorsed by the publisher.
